# Secundum atrial septal defect in adults: a practical review and recent developments

**DOI:** 10.1007/s12471-015-0663-z

**Published:** 2015-03-04

**Authors:** Joey M. Kuijpers, Barbara J.M. Mulder, Berto J. Bouma

**Affiliations:** 1 Department of Cardiology, Academic Medical Center, University of Amsterdam, Amsterdam, The Netherlands; 2 Interuniversity Cardiology Institute of the Netherlands – Netherlands Heart Institute, Utrecht, The Netherlands

**Keywords:** Secundum atrial septal defect, Adult, Diagnosis, Transcatheter closure, Surgery

## Abstract

Secundum atrial septal defect (ASDII) is a common congenital heart defect that causes shunting of blood between the systemic and pulmonary circulations. Patients with an isolated ASDII often remain asymptomatic during childhood and adolescence. If the defect remains untreated, however, the rates of exercise intolerance, supraventricular arrhythmias, right ventricular dysfunction and pulmonary arterial hypertension (PAH) increase with patient age, and life expectancy is reduced. Transcatheter and surgical techniques both provide valid options for ASDII closure, the former being the preferred method. With the exception of those with severe and irreversible PAH, closure is beneficial to, and thus indicated in all patients with significant shunts, regardless of age and symptoms. The symptomatic and survival benefits conferred by defect closure are inversely related to patient age and the presence of PAH, supporting timely closure after diagnosis. In this paper we review the management of adult patients with an isolated ASDII, with a focus on aspects of importance to the decision regarding defect closure and medical follow-up.

## Introduction

Patients with an isolated secundum atrial septal defect (ASDII) have benefited from diagnostic and therapeutic advancements over the past decades. They can be diagnosed and treated early in life, but frequently remain asymptomatic and unrecognised well into adulthood. Consequently, these patients form a heterogeneous adult population regarding disease burden, risk of complications and required medical surveillance and treatment. In this paper we review the diagnosis and management of ASDII in adults, with attention to recent developments. We focus on the aspects relevant to the decision regarding defect closure, its benefits and limitations and the necessity for continued follow-up, that are important to the general cardiologist confronted with and caring for these patients.

## Prevalence

Atrial septal defect (ASD) is a common congenital heart defect, with an estimated birth prevalence of 1.6 per 1000 live births and a 97 % probability of survival into adulthood [1, 2]. ASDII constitutes about 75 % of these defects, has a female predominance of approximately 2:1, and is frequently diagnosed in adulthood [3, 4].

## Genetic factors

Several genetic syndromes, such as Down, Holt-Oram and Noonan, are associated with ASDII [5]. However, most secundum ASDs occur sporadically, complicating identification of possible causal genes. Nevertheless, several genes underlying ASDII have been identified, including transcription (co-)factor encoding genes such as *GATA4*, *NKX2.5*, and *TBX5,* and sarcomeric protein encoding genes such as *ACTC1*, *MYH6* and *MYH7*. Secundum ASDs associated with atrioventricular block are linked to *NKX2.5* mutations [6, 7]. Although a proportion of secundum ASDs are thus attributable to single-gene defects, its generally sporadic occurrence underlines a multifactorial causal mechanism, involving multiple susceptibility genes and environmental factors [8]. Nevertheless, with familial occurrence of ASDII, cardiogenetic testing is worth consideration as this might identify a known gene mutation, prompt evaluation of and earlier diagnosis in relatives, and aid genetic counselling.

## Pathophysiology

The direction and magnitude of flow through an ASDII depends on defect size and the relative compliance of the left and right heart chambers. Initial left-to-right shunting results from the greater compliance of the right ventricle and right atrium, relative to the left heart chambers. A haemodynamically significant shunt causes right-sided volume overload [9]. Resultant right ventricular (RV) enlargement shifts the interventricular septum toward the left ventricle during diastole. This impairs left ventricular (LV) filling, leading to reduced LV stroke volume and cardiac output, and increased left-to-right shunting [10]. A longstanding shunt results in reduced RV compliance, increased right-sided pressures and reduced left-to-right shunting. This can culminate in RV failure. Pulmonary vascular disease and pulmonary arterial hypertension (PAH) develop in a minority of patients, predominantly in females. Its aetiology is likely multifactorial, and not dependent on shunt size and duration alone. Eventually, pulmonary pressures can reach (supra)systemic levels, causing reversal of the intracardiac shunt: Eisenmenger physiology [3, 11].

## Natural history and presenting symptoms

Patients with an isolated ASDII often remain asymptomatic during childhood and adolescence. However, most will become symptomatic from the third or fourth decade, and life expectancy is reduced overall [12]. Common initial symptoms are exercise intolerance and fatigue, which may be aggravated by a supraventricular tachycardia (SVT). SVTs are not infrequently the first clinical manifestation of an ASDII in patients over 40 years [13]. Eventually, right-sided heart failure can develop, often with mild to moderately elevated pulmonary arterial pressure (PAP) [12, 14]. Severe PAH, with possible progression to Eisenmenger physiology, ensues in a minority of patients [11]. Occasionally, a suspected paradoxical systemic thromboembolism initially raises the suspicion of an ASDII being present [15].

## Diagnostic work-up

**Fig. 1:**
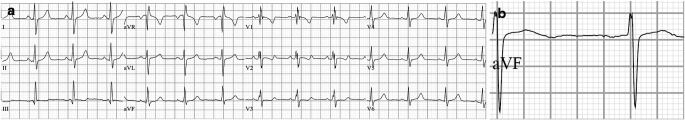
**a** ECG of a 38-year-old woman with an ASDII. Mild right-axis deviation, rsR’ pattern in lead V1 reminiscent of a partial right bundle branch block. There is no further evidence of right ventricular hypertrophy. **b** Detail of lead aVF from an ECG of a 31-year-old man with an ASD, showing a notch on the R wave: crochetage

Key findings of RV volume overload are RV heave, wide and fixed splitting of the second heart sound and a systolic pulmonary flow murmur. The ECG typically shows an rsR’ pattern in the right precordial leads and right QRS-axis deviation. Both reflect RV hypertrophy. An early notch on the R wave in the inferior leads, called crochetage, is 92–100 % specific for ASDII when present in all three leads (Fig. 1) [16]. The *chest X-ray* may show enlargement of the right heart chambers and both central and peripheral pulmonary vasculature.

*Transthoracic echocardiography* (TTE) is the primary imaging modality for the diagnosis of an ASDII and assessing its haemodynamic consequences. RV volume overload, reflected by RV enlargement and diastolic flattening and paradoxical systolic movement of the interventricular septum, indicates a significant shunt [10]. RV and PA pressures are estimated from the peak tricuspid regurgitation jet velocity. When TTE is inconclusive, contrast echocardiography can confirm the presence of an interatrial shunt. *Transoesophageal echocardiography* (TEE) is required for determining the feasibility of transcatheter closure (See *‘Closure strategy and post-procedural follow-up’* below) [17]. Referral to an adult congenital heart disease (ACHD) centre for further imaging is indicated when interatrial shunting or RV overload is recognised, but cannot be explained.

*Cardiac magnetic resonance imaging* (MRI) can help clarify the morphology of the defect, and is the gold standard for measurement of ventricular volume and function [18, 19]. *Cardiac computed tomography* (CT) provides an alternative in patients with contraindications to MRI [20]. *Diagnostic catheterisation* is only indicated when the possibility of closure is uncertain in patients with high estimated PAP on echocardiography, to assess (reactivity of) pulmonary vascular resistance (PVR) [9, 11].

## Defect closure

### Indications and contraindications for defect closure

**Fig. 2:**
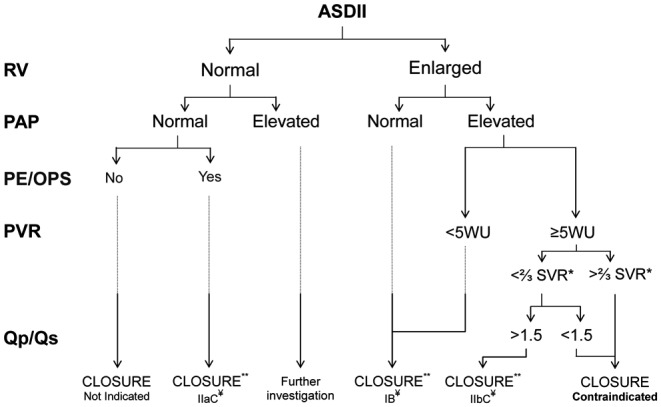
Flow diagram of the factors involved in the decision concerning defect closure in adults with an ASDII. *RV* right ventricle, *PAP* pulmonary artery pressure, *PE* paradoxical embolism, *OPS* orthodeoxia-platypnoea syndrome, *PVR* pulmonary vascular resistance, *Qp/Qs* pulmonary-to-systemic flow ratio, *WU* Woods unit, *SVR* systemic vascular resistance. *either at baseline or after pulmonary vasodilator challenge or targeted pretreatment. **unless severe left ventricular dysfunction and/or mitral insufficiency are present. *¥* class of recommendation and level of evidence

Unless severe and irreversible PAH is present, patients with a haemodynamically significant shunt (i.e. one that causes RV enlargement) should undergo ASDII closure, irrespective of age and symptoms (Fig. 2) [21–23]. Haemodynamically insignificant ASDs do not require closure. Such patients should be followed conservatively, with repeat echocardiography every 2–3 years, as shunt size may increase due to reduction in LV compliance associated with systemic hypertension and/or coronary artery disease. When a paradoxical embolism is suspected, or the rare orthodeoxia-platypnoea syndrome is documented, closure is indicated regardless of shunt size [9, 15, 24].

PAH is not an absolute contraindication for defect closure [3]. Although estimation of PAP on echocardiography is usually sufficient, a diagnostic catheterisation to determine (reactivity of) PVR is indicated in selected patients. Closure is indicated if PVR is < 5 Wood units (WU). If the PVR is ≥ 5 WU, closure can still be considered if the pulmonary-to-systemic flow ratio is > 1.5 and PVR or PAP is less than two-thirds of the systemic levels or reactive to a pulmonary vasodilator challenge or targeted pretreatment. Patients with severe and irreversible PAH are not eligible for closure, as the ASDII is then physiologically needed for RV decompression. Similarly, defect closure is contraindicated in case of significant LV dysfunction, as it then decompresses the LV. Thus, patients with PAH or poor LV function should be evaluated in ACHD centres, as they may require invasive pre-interventional testing and treatment [9].

### Closure strategy and post-procedural follow-up

*Surgical repair* of secundum ASDs is an effective procedure with practically no perioperative mortality and minimal morbidity, although the latter is somewhat higher in elderly patients [25]. Pericardial or synthetic patch closure is preferred over a direct suture, and generally completely terminates the shunt. Minimally invasive techniques have improved cosmetic outcomes, while maintaining the safety and efficacy of the traditional sternotomy [26]. Postoperative follow-up should include ECG surveillance for SVTs, and echocardiographic assessment of residual shunting, RV size and function and PAP. Subsequent annual follow-up is recommended for patients with an ASDII repaired in adulthood, who have or develop SVTs, PAH, ventricular dysfunction or valvular lesions. Current guidelines state that regular follow-up is not required for patients repaired under the age of 25 without relevant complications or residuae [9]. However, recent studies, and yet unpublished results from our own institution, show that even patients with early closure remain at risk of developing PAH late after closure [4, 27, 28]. This indicates that at least sporadic follow-up (i.e. once every 5 years) is indicated in all patients with a closed ASDII.

**Fig. 3:**
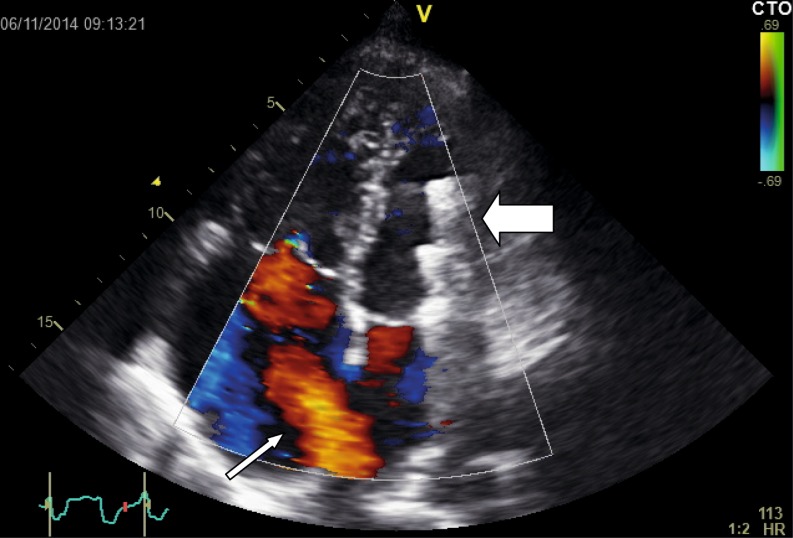
Transthoracic echocardiogram from a patient with an ASDII, showing clear left-to-right shunting through an open defect (*small white arrow*) and right-atrial enlargement. The previously implanted closure device has embolised to the left ventricle (*large white arrow*)

*Transcatheter device closure* is generally the treatment of choice nowadays. It provides similar efficacy and haemodynamic benefits, but reduced complication rates and duration of hospital stay compared with surgery, especially in older patients [29]. Secundum ASDs > 38 mm in diameter, those with inadequate septal rims for device anchorage, and those in which the device would interfere with atrioventricular valve function or venous drainage are not eligible and referred for surgical closure [17]. In selected adult patients, the closure rate of single defects is 96 %, and occurrence of major periprocedural complications is < 1 % [30]. Endocarditis prophylaxis is required during the first 6 months after device closure, as is antiplatelet therapy (aspirin 100 mg) [31]. Although rare, late complications, including device embolisation, erosion through the atrial wall or aortic root and obstruction of venous drainage, necessitate follow-up (Fig. 3) [32]. However, consensus regarding frequency and duration of follow-up after device closure is lacking. It seems prudent to follow all adult patients regularly during the first year (at 1, 6 and 12 months), and periodically thereafter. Those treated after the age of 40, and those with residual shunts, elevated PAP or documented dysrhythmias should have regular follow-up at an ACHD centre for 2 years, and every 2–4 years thereafter [9]. As stated above, all patients should probably remain under follow-up.

**Fig. 4:**
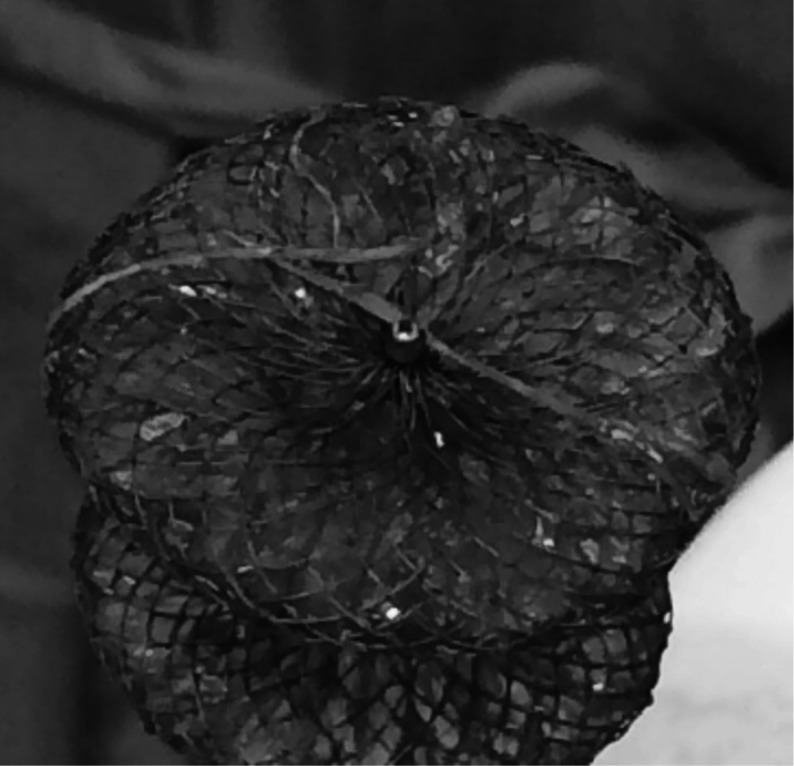
Synthetic ASDII closure device, the Amplatzer Septal Occluder (AGA Medical Corporation, Plymouth, MN), which was retracted after attempted implantation. The metal wires and synthetic meshwork are clearly seen. Entangled in the device is the Chiari network, which hampered device deployment

The most commonly used devices, composed of synthetic material and metallic wires (Fig. 4), induce fibrous encapsulation and neo-endothelialisation that eventually renders them functionally obsolete. However, the risk of long-term device-related complications such as erosion, device embolisation and nickel allergy remains [32]. Furthermore, left atrial access for treatment of atrial dysrhythmias or mitral valve disease might be obstructed, although this is not necessarily an issue (See *‘Supraventricular tachycardias’* under *‘Effects of defect closure’* below). The biodegradable BioSTAR implant (NMT Medical, Boston, MA) consists of collagen on a metal framework, is almost entirely absorbed after tissue overgrowth and thus potentially reduces the risk of late device-related complications. Early results indicate safe and effective ASDII closure can be achieved with this device [33]. However, recent results indicate a high residual shunt rate (30 %) after 2 years [34]. Whether the potential for complications is indeed reduced, and what the place of biodegradable devices in ASDII treatment should be, remains to be determined.

### Effects of defect closure

*Cardiac remodelling and exercise capacity*. Closure of the ASDII results in right-sided volume unloading and reduction in RA and RV size. The remodelling process and associated increase in cardiopulmonary function commence immediately after closure and continue for several years [10, 35, 36]. Decreased RV volume improves ventricular interaction and LV filling. Subsequent increase in LV stroke volume and cardiac output is probably the main mechanism behind the improvement of exercise capacity after closure. These effects occur in patients of all ages, both symptomatic and asymptomatic. Although normal exercise capacity is reached in the majority, it may remain subnormal in those with poor cardiopulmonary function prior to closure [10]. This supports timely closure of sizeable secundum ASDs, regardless of age and symptoms.

*Pulmonary hypertension.* After ASDII closure in childhood or adolescence, PAH development later in life is unlikely, although not completely obviated [4, 27, 28, 37]. Among untreated adults, older age, greater defect size and female sex are associated with a greater frequency of PAH. Provided that pulmonary vascular disease has not progressed irreversibly, device closure can safely and effectively reduce PAP. Higher PAP and younger age are associated with a greater chance and magnitude of reduction in PAP. However, normalisation of PAP occurs less frequently with more severe PAH [3]. Moreover, if closure is performed late in life, the chance of PAH developing thereafter is increased [4]. These data strengthen the rationale for early closure.

Patients with an ASDII, open or closed, and PAH should be referred to an ACHD centre. As their haemodynamic balance is readily disrupted, planned medication changes or interventions should be discussed with their specialised caregiver. Moreover, the prescription of disease-targeting therapy such as the endothelin antagonist bosentan is restricted to these specialists. When PAH develops late after closure, other non-shunt-related risk factors should be ruled out to appropriately target therapy [9, 11].

*Supraventricular tachycardias* are common late in the natural history of an ASDII [13, 21], and defect closure is of preventive, but not therapeutic value in this regard. Thus, SVTs are not a decisive indication for defect closure in the absence of other symptomatology. Among patients who underwent closure in childhood or early adolescence, SVTs are rare during long-term follow-up [37]. Those with a history of SVT or PAH and those treated later in life, especially at ages over 40 years, remain at increased risk [13, 21, 38]. Paroxysmal SVTs may not recur after closure, but chronic SVTs usually persist. Concomitant Maze procedure during surgery or electrical cardioversion at the time of device closure is worth consideration [9, 38, 39]. Class III antiarrhythmic agents may be the most effective in maintaining sinus rhythm after cardioversion in these patients [40].

In patients developing symptomatic drug-resistant atrial fibrillation after closure, catheter ablation is an effective treatment. Perceived difficulty in obtaining prerequisite left atrial access may discourage its utilisation in patients with an ASD closure device. However, intracardiac echocardiography-guided transseptal catheter ablation is safe and effective, even in the presence of a closure device. Left atrial access is generally achieved through residual native septum. In the absence of a suitable native septal area, direct puncture of the Amplatzer device is possible, at the cost of greater technical difficulty and longer procedural time. Although no long-term data are available, complications and residual shunts are rare with both approaches [41]. Retrograde aortic access using remote magnetic navigation may provide an alternative approach [42].

*Survival.* Closure confers a survival benefit in all ASDII patients, regardless of age [22]. However, eventual life expectancy is dependent on age at closure. An often-cited study into the long-term outcome after surgical repair showed normal survival after closure under the age of 25. In contrast, patients operated later in life, especially at ages over 40, experienced increased mortality [21]. Percutaneous closure can be expected to confer a survival benefit at least similar to that reported in surgical series, but a randomised comparison to prove and quantify this benefit would be unethical in the current era. The excellent survival of the contemporary ASDII population is confirmed by unpublished data from our own institution which suggest normal overall life expectancy, although this appears to be restricted to the female majority [43, 44].

## Sports participation, diving and high altitude

The basis for recommendations regarding the safety of competitive sports participation is the individual patients’ abilities, haemodynamics and risk of decompensation or dysrhythmias. Nevertheless, general recommendations can be given. Evaluation should include a comprehensive history and physical examination, ECG, chest X-ray, TTE and exercise testing. Before or from 6 months after defect closure, asymptomatic patients with normal PAP can participate in all sports, while restriction to low-intensity sports is indicated when PAH is present. Eisenmenger physiology precludes sports participation. In the presence of symptomatic dysrhythmias, second-degree or third-degree heart block or ventricular dysfunction, individual evaluation and exercise prescription are indicated, for which we refer to the current guidelines [45].

Scuba diving carries the risk of developing decompression illness (DCI), which results from gasses coming out of solution into bubbles in blood and tissue upon ascent from a dive. Although absolute risk remains low, divers with an interatrial communication are at increased risk for major neurological DCI [46]. The presumed mechanism is paradoxical embolisation of venous gas bubbles to the systemic arterial circulation. The association between interatrial shunts and neurological DCI has mainly been studied in divers with a patent foramen ovale, but the same principles apply to ASDII. Defect closure might prevent the occurrence of neurological DCI in divers, but this was shown in only one small prospective study [47]. Based on the above, screening for an interatrial shunt may be indicated in divers that have developed DCI. When present, closure of the interatrial defect may prevent occurrence of major DCI, but the evidence is scarce and abstinence from diving might be advisable.

Regardless of health status, residing at high altitude exposes the individual to lower environmental partial oxygen pressures and lower arterial oxygen tensions, with the risk of maladaptation and height-related diseases. These issues should be addressed in pretravel counselling [48]. Evidence-based recommendations for stays at high altitude in adult ASDII patients are not available. However, patients with uncomplicated defects should not require additional assessment. In contrast, patients with PAH or cyanosis require echocardiographic assessment of RV function and PAP, as well as cardiopulmonary exercise testing. At high altitude, hypoxic pulmonary vasoconstriction raises PVR resulting in persistent elevation of PAP. Consequently, RV workload and right-to-left shunting increase, predisposing these patients to RV failure, severe hypoxaemia and the development of high altitude pulmonary oedema [48]. Cyanotic patients with coexisting anaemia, ventricular dysfunction or low exercise capacity should avoid stays at high altitude (> 1800 m), as should those with PAP > 35 mmHg. If they do travel to such altitudes, supplemental oxygen is to be advised. Pharmacological prophylaxis (nifedipine, sildenafil) can be considered for those with milder PAH. The evidence upon which these recommendations are based has been reviewed by Luks et al. [49].

## Pregnancy

Women with an open ASDII not complicated by PAH generally tolerate pregnancy well. Defect closure before pregnancy, however, might lower the risk of paradoxical embolus and functional deterioration. Moreover, compared with the general population, women with an open ASD have increased risks of pre-eclampsia, foetal loss and low birth weight. In contrast, outcome for women with a closed ASD is similar to that of the general population [50]. Thus, if the diagnosis has been established, closure of the defect before pregnancy is preferable.

Pregnancy is contraindicated in women with severe PAH, especially in those with Eisenmenger physiology. Patients and partners should be counselled about avoiding pregnancy. Referral to a high-risk obstetrical specialist is recommended for selecting contraceptive methods, as all carry specific risks [9].

## Conclusions

Patients with an ASDII that causes enlargement of the right heart chambers are subject to important age-related morbidity and reduced life-expectancy. All haemodynamically significant secundum ASDs should be closed, regardless of age and symptoms, preferably using a transcatheter device. Current evidence suggests the beneficial effects of defect closure to be inversely related to patient age, supporting timely closure after diagnosis. Treated patients, especially those with pre-existing PAH or dysrhythmia and those treated later in life, remain at increased risk for cardiovascular morbidity and should stay under medical surveillance.
